# GISA: using Gauss Integrals to identify rare conformations in protein structures

**DOI:** 10.7717/peerj.9159

**Published:** 2020-06-11

**Authors:** Christian Grønbæk, Thomas Hamelryck, Peter Røgen

**Affiliations:** 1 Department of Biology, University of Copenhagen, Copenhagen, Denmark; 2 Department of Biology, Department of Computer Science, University of Copenhagen, Copenhagen, Denmark; 3 DTU COMPUTE, Technical University of Denmark, Kgs. Lyngby, Denmark; 4 Current affiliation: Department of Biology, Novo Nordisk Foundation Center for Basic Metabolic Research, University of Copenhagen, Copenhagen, Denmark

**Keywords:** Protein structure analysis, Knots and links, Gauss integrals, Rare conformations, Sub-chains, Fast algorithm, Database scan, Topology, Geometry

## Abstract

The native structure of a protein is important for its function, and therefore methods for exploring protein structures have attracted much research. However, rather few methods are sensitive to topologic-geometric features, the examples being knots, slipknots, lassos, links, and pokes, and with each method aimed only for a specific set of such configurations. We here propose a general method which transforms a structure into a ”fingerprint of topological-geometric values” consisting in a series of real-valued descriptors from mathematical Knot Theory. The extent to which a structure contains unusual configurations can then be judged from this fingerprint. The method is not confined to a particular pre-defined topology or geometry (like a knot or a poke), and so, unlike existing methods, it is general. To achieve this our new algorithm, GISA, as a key novelty produces the descriptors, so called Gauss integrals, not only for the full chains of a protein but for all its sub-chains. This allows fingerprinting on any scale from local to global. The Gauss integrals are known to be effective descriptors of global protein folds. Applying GISA to sets of several thousand high resolution structures, we first show how the most basic Gauss integral, the writhe, enables swift identification of pre-defined geometries such as pokes and links. We then apply GISA with no restrictions on geometry, to show how it allows identifying rare conformations by finding rare invariant values only. In this unrestricted search, pokes and links are still found, but also knotted conformations, as well as more highly entangled configurations not previously described. Thus, an application of the basic scan method in GISA’s tool-box revealed 10 known cases of knots as the top positive writhe cases, while placing at the top of the negative writhe 14 cases in cis-trans isomerases sharing a spatial motif of little secondary structure content, which possibly has gone unnoticed. Possible general applications of GISA are fold classification and structural alignment based on local Gauss integrals. Others include finding errors in protein models and identifying unusual conformations that might be important for protein folding and function. By its broad potential, we believe that GISA will be of general benefit to the structural bioinformatics community. GISA is coded in C and comes as a command line tool. Source and compiled code for GISA plus read-me and examples are publicly available at GitHub (https://github.com).

## Background

Røgen & Bohr (2003) introduced a set of quantitative protein fold descriptors consisting in 29 knot-theoretic Gauss Integral (GI) based invariants, shown shortly after in Røgen & Fain (2003) to be able to automatically recover the classification of the CATH database.

Automated *local* scrutiny of folds is desired for various purposes, including identifying odd shapes in predictions, improving classification and for structure alignments. However, while the GI invariants work very well as *global* fold descriptors, an efficient method for computing them locally has been lacking and local applications have been few. Revealing though indeed the relevance of applying GIs locally, the structural similarity methods in Chang, Rinne & Dewey (2006) and Zhi, Shatsky & Brenner (2010) based on local writhe showed excellent performance (writhe is an order one GI). Due to its recursive nature, our new algorithm, GISA, computes not only the GI invariants of an entire chain but at the same time of all sub-chains, allowing therefore structural analyses on any scale from local to global (we assume chains and sub-chains to be connected).

By the knot-theoretic nature of the GIs, GISA is sensitive to topologic-geometric differences, while having the fundamental translational-rotational invariance. This distinguishes GISA from distance based approaches. A general method for structural analysis having such topologic-geometric sensitivity seems still to be lacking (Jarmolińska et al., 2018; Marks et al., 2011). By its versatility, we believe that GISA can fill this gap.

As a tool GISA includes computation of the desired GIs, deriving GI-values for sub-chains and search/scan methods. The latter allow to rank a set of query structures’ GI-values against a background, consisting in a set of GI-values, likewise produced by running GISA on a “data base” of structures.

The main aims of this paper are to introduce GISA, to explain the method via a proof-of-concept and describe the contents of the tool. The primary focus is on the proof-of-concept, which only involves the lowest order GI, viz. the writhe: First we show in a “restricted search” how GISA can be exploited to provide an algorithm for identifying particular geometries in folds such as a chain forming an almost closed loop through which it passes, or two such loops interlinking (these were termed “pokes” and “co-pokes”, respectively, by  Khatib, Rohl & Karplus (2009)). Then, in a similar unrestricted search, we show how GISA allows identifying the very same configurations as well as more elaborate ones, by letting the search be based on finding outlying writhe values only (please see the 3d-figures below; more examples from both searches can be found in Supplemental Information 1).

Recent work by Dabrowski-Tumanski & Sulkowska (2017) indicates the steady interest in interlinking loops and closely related topologies. While search algorithms as those in Dabrowski-Tumanski & Sulkowska (2017), Khatib, Rohl & Karplus (2009), Niemyska et al. (2016) build on predefined shapes, our algorithm searches for exceptional values of GIs, and the shapes of the search hits are then final output rather than input; thus GISA allows to identify both less constrained as well as more elaborate shapes. This we support by running the unrestricted search and, more generally, by the runs of GISA’s basic scan method, rar0. This scan tool formalizes the unrestricted search (rar is short for rarity). It allows assessing a set of structures on a larger background by means of writhe values only (GISA has two additional scan tools that allow using higher order GIs).

Our proof-of-concept—the restricted and unrestricted searches—and rar0 make use only of the writhe for identifying locally entangled configurations. Several authors have recently made similar use of the writhe, but for defining a *global* entanglement of one or more polymers (Baiesi et al., 2016; Baiesi et al., 2017; Baiesi et al., 2019; Panagiotou & Kröger, 2014; Panagiotou, Kröger & Millett, 2013; Panagiotou & Plaxco, 2019; Panagiotou, Millett & Atzberger, 2019). A general aim there is to relate such an entanglement measure to physical properties of the molecules. In these works the writhe is applied to open curves (Baiesi et al., 2016; Panagiotou & Kröger, 2014; Panagiotou, Kröger & Millett, 2013; Panagiotou & Plaxco, 2019; Panagiotou, Millett & Atzberger, 2019). Closest to our application of the local writhe numbers are Baiesi et al. (2017); Baiesi et al. (2019). These are however restricted to the particular geometry consisting in pokes (threads) of almost closed loops with no restriction on the length of the poking segment except a lower bound of 10 residues (which coincidentally is the *largest* poke-length we consider). The “maximal poke writhe-value” is then used as the global measure.

In another direction, well-established knot-invariants (“knot polynomials”) have been applied to detecting knots in proteins. A particular case is that of slipknots (King, Yeates & Yeates, 2007), where particular care is taken in closing the sub-chains. A more general approach is that of knotoids Goundaroulis et al. (2017), which allows similar detection of knots while working on the (open) chain, i.e., even without performing random closures of it (see also Dabrowski-Tumanski et al., 2018). The method is though still stochastic in nature.

The other main tools in GISA are the GI-generating functions and two more advanced scan methods (rar1 and rar2). All three scan tools build on the possibility of pre-computing the GIs so as to generate a background against which query sets can be swiftly assessed. Key in the pre-computability of the GIs is their translational and rotational invariance (no costly superpositions of the structures are needed). Since our focus in this paper is on the application of the writhe, we dedicate most time to the basic rarity scan, rar0. The strength of the two more advanced scan flavours, rar1 and rar2, is that they allow including higher order GIs. We include a short description below; more can be found in Supplemental Information 1.

To validate the output of the restricted and unrestricted searches we visually inspect the cases having the most conspicuous writhe values and compare their rankings. To assess the validity of GISA’s scan methods, we check that the top-rankings of the basic scan tool, rar0, match those of the unrestricted search. As for rar1/2 we show that their outputs are well-aligned at similar settings (Supplemental Information 1).

The command line tools (compiled code) as well as the source C code for computing the invariants up to and including order 3, for supporting the scans and for the particular searches are available at GitHub under GNU General Public License v3 (https://github.com/ceegeeCode/GISA).

## Methods

We represent a protein chain by the piece-wise linear curve given by its *α*-Carbon trace. A poke consists of an almost closed loop (of moderate length) through which a shorter segment of the chain sticks—or pokes. A co-poke can be understood as a “1-link”: two almost closed loops (of moderate length) poking through each other, i.e., interlinking once. For an example of a poke see Fig. S15; examples of 1-links can be seen in the 3d-figures right below.

In a 1-link the two smooth closed loops have a linking number, or writhe, of ± 1. The writhe is therefore a key notion in this paper why we give a short introduction now.

### A primer on Writhe

Writhe is a geometric measure of how coiled a space curve is. Consider the carbon alpha curve of a protein chain, or a fragment thereof, traversed in the N-to-C direction. Look at the curve from one direction in space, or mathematically project the curve onto a plane, and keep track of over and under crossings. A crossing is called positive if the directions of traversal at the crossing follow the right hand rule of electro-magnetic induction; otherwise, it is called negative. The *directional* writhe is simply the sum of the signs of the crossings seen from a given direction. This sum is a natural notion of how coiled the planar curve with over and under crossings is. The number of crossings and their signs may change if you observe the curve from another direction. Hence, the directional writhe in general depends on the chosen direction. By averaging the directional writhe over all directions, we get the average signed number of crossings or writhe of the curve. The writhe is by the averaging independent of the curve’s position in space—the writhe is invariant under rotations and translations of the curve.

For two closed non-intersecting curves that both have a chosen direction of traversal the directional writhe can be shown to be constant, i.e., independent of the chosen projection. This constant is called the linking number and counts how many times the two curves are linked together. If however the curves are only almost closed, the chosen direction matters. In this case we can though still apply the writhe and, loosely speaking, the writhe increases (in absolute value) the more the two curves interlink. In case the two curves are closed the writhe and the directional writhe will be equal. We can then still consider the writhe as a measure of the winding. Writhe or linking number is most effectively calculated using a so-called Gauss Integral (for more see Røgen & Bohr, 2003).

Supplemental Information 1 contains a detailed walk-through of our application of the writhe and what GISA computes in the case of the protein 1bpi, which contains a 1-link (see the 3d-figure right below).

### Approach in restricted and unrestricted searches

As we have now seen, if one can compute the writhe values of all pairs of (almost) closed loops in a chain, instances of (almost) 1-links can possibly be identified (cases of higher linking numbers—i.e., one loop winding around the other several times—are less expected due to their lower entropy). A major benefit of our GISA algorithm is that it allows exactly that: it computes not only Gauss Integrals of the whole chain, but of all sub-chains. This allows computing swiftly the desired linking number—or *mutual writhe* value—of any given pair of sub-chains (Supplemental Information 1, Eq. 3).

Pokes can be searched for similarly by considering the mutual writhe values of short sub-chains (length 10, say) vs. (almost) closed loops. The intuition here is from electromagnetism: For a line segment placed in the magnetic field induced by an electric current in a wire-loop, the change in magnetic potential along the segment is larger the “purer” it pokes the loop.

The base of GISA consists in an algorithm computing the GIs, which includes also support for the two modes: 1) for the restricted search, all (almost) closed loops are identified, where after the relevant mutual writhe values for link/poke searching are derived and written to file; 2) for the unrestricted search, the algorithm simply derives the mutual writhe of all pairs of sub-chains of some fixed length. To limit the size of the output, we keep only the pairs of sub-chains with the lowest and with the highest mutual writhe values (a similar limitation of the output is made for the pokes of the restricted search). The derivation of the mutual writhe amounts to adding four look-ups in the stored writhe-table. In both versions GISA returns the identified sub-chain pairs along with their mutual writhe. Conspicuous cases are then found in the tails of the resulting distribution of mutual writhe values. In the restricted search we expect to find 1-links/pokes and, when using the unrestricted search, possibly other geometries as well.

While a small post-processing task is here needed to identify tail events, GISA’s scan methods that we now turn to directly rank structures in terms of their “GI content”. Of these the basic method (rar0) is the offspring of our proof-of-concept.

### GISA’s scan methods

As explained GISA includes tools for querying possible rarity in a set of structures against a preferably larger and representative set of structures. The output is a score and a probability/rank for each queried structure, with all queried structures listed by decreasing score (and increasing probability/rank). GISA has three types of scans to support this, rar0, rar1 and rar2 (in the command line tool these are referred to as flavours). In all three, sub-chains (windows) of a user-defined length are considered , covering each structure by moving the window along the structure at a likewise user-defined step size.

The basic scan method (rar0) assesses each structure by its content of mutual writhe above a set threshold (there are two versions of rar0, see Supplemental Information 1; results below are found using version A).

While rar0 is based only the single value of the mutual writhe, the two other types of scans, rar1 and rar2, can use higher order GIs. Both are therefore based on arrays of GIs. The scan approach builds on discretizing the GI-arrays and looking up the resulting GI-words in the likewise “dictionaried” background of GI-arrays. This can be seen as local structural alignment in the space of GI-arrays (rather than in 3d-space) and could serve as the foundation of a similar global structural alignment. More details on each scan method can be found in Supplemental Information 1.

### Data and implementation

For the bulk of our analyses we use the top100 and top8000 sets available on the Kinemage homepage (Kinemage, 2016), consisting of respectively 100 and about 8,000 high resolution protein structures in PDB format (PDB, 2016). For a more recent data set we consider the Pisces lists “cullpdb _pc20 _res3.0 _R1.0 _d200123” and its high resolution subset “cullpdb _pc20 _res1.8 _R0.25 _d200115” (Dunbrack Lab, 2020). We refer to these sets as PiscesLoRes and PiscesHiRes, respectively.

All results are made by compiling and running the C code on a unix server, except for the computational performance (Supplemental Information 1) which was made with a Windows-compiled version of the code run on a common laptop (Intel Core i7-4510, 2.00 GHz/2.60 GHz, 8GB RAM, hard disc of SSD type; OS Microsoft Windows 10).

The C source code along with implementation notes can be found in the Github repository, which also contains outlines of the code for the key functions for GISA’s tools and examples on how to run the code. The Python code and Pymol scripts for plotting and selecting examples for the restricted and unrestricted searches in this paper are also placed in the repository. We have also placed html-code for the shown structures/examples in the Github repository. This is based on the NGL viewer (Rose & Hildebrand, 2015; Rose et al., 2018). Opening one of these files in a standard internet browser yields an interactive plot of the given structure (NGL viewer).

## Results

### Restricted and unrestricted searches

For the restricted search we follow Khatib, Rohl & Karplus (2009) and let a closed loop mean a sub-chain consisting of no less than 6 and no more than 30 line-segments and such that the *α*-Carbon atoms at the sub-chain terminals are at most 7 Ångström apart.

For the unrestricted search we consider sub-chains of length 30 (results for length 15 are given in Supplemental Information 1). For both types of search we consider only pairs of non-overlapping sub-chains. More examples can be found in Supplemental Information 1.

**Figure 1:**
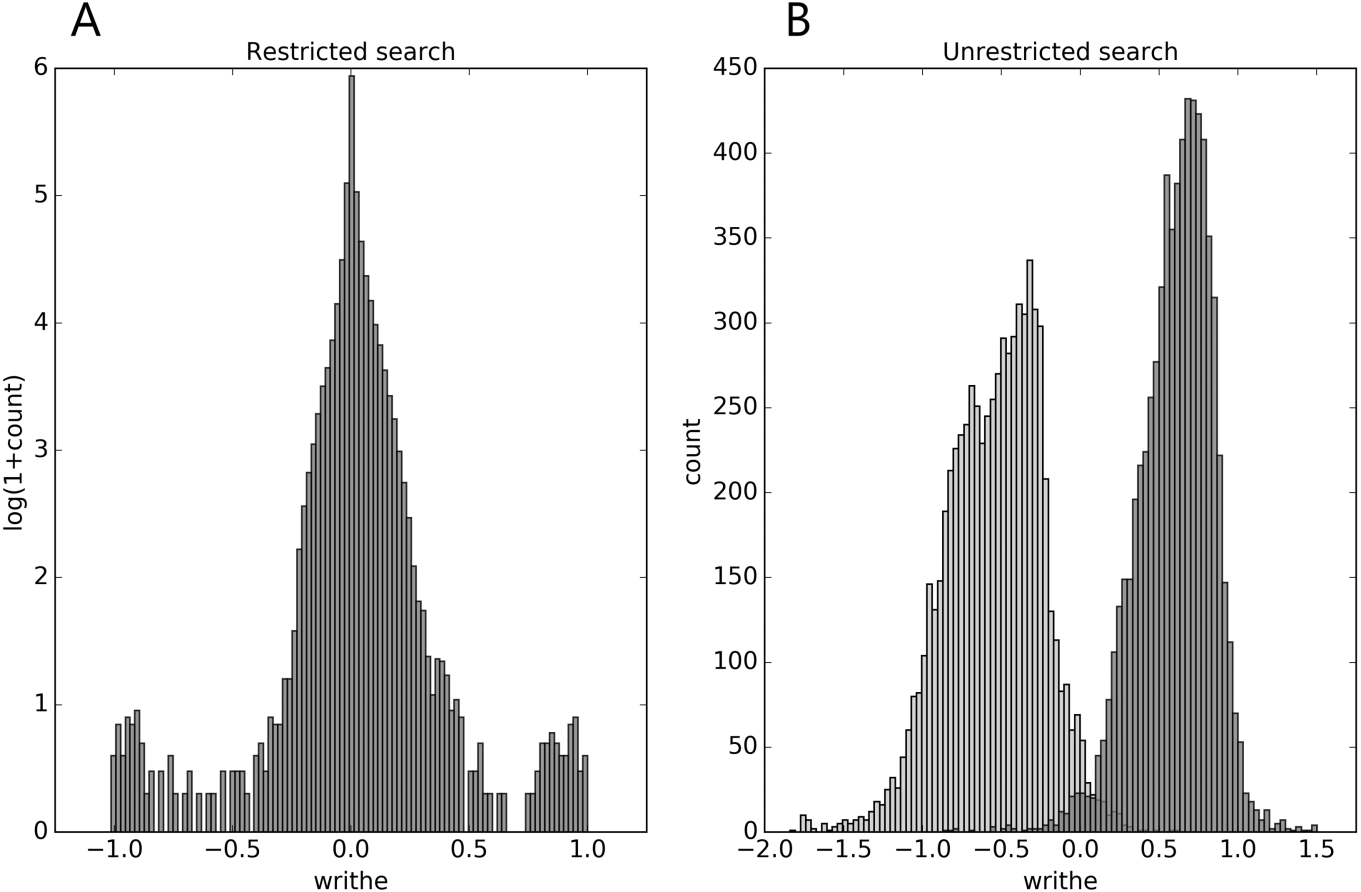
Distributions of mutual writhe values for potential links (A, restricted search) and for pairs of sub-chains of length 30 (B, unrestricted search) throughout the top8000 set, obtained as described in the text. For the unrestricted search the light-grey (dark-grey) bars show the distribution of the lowest (highest) writhe value per chain (see text).

**Figure 2:**
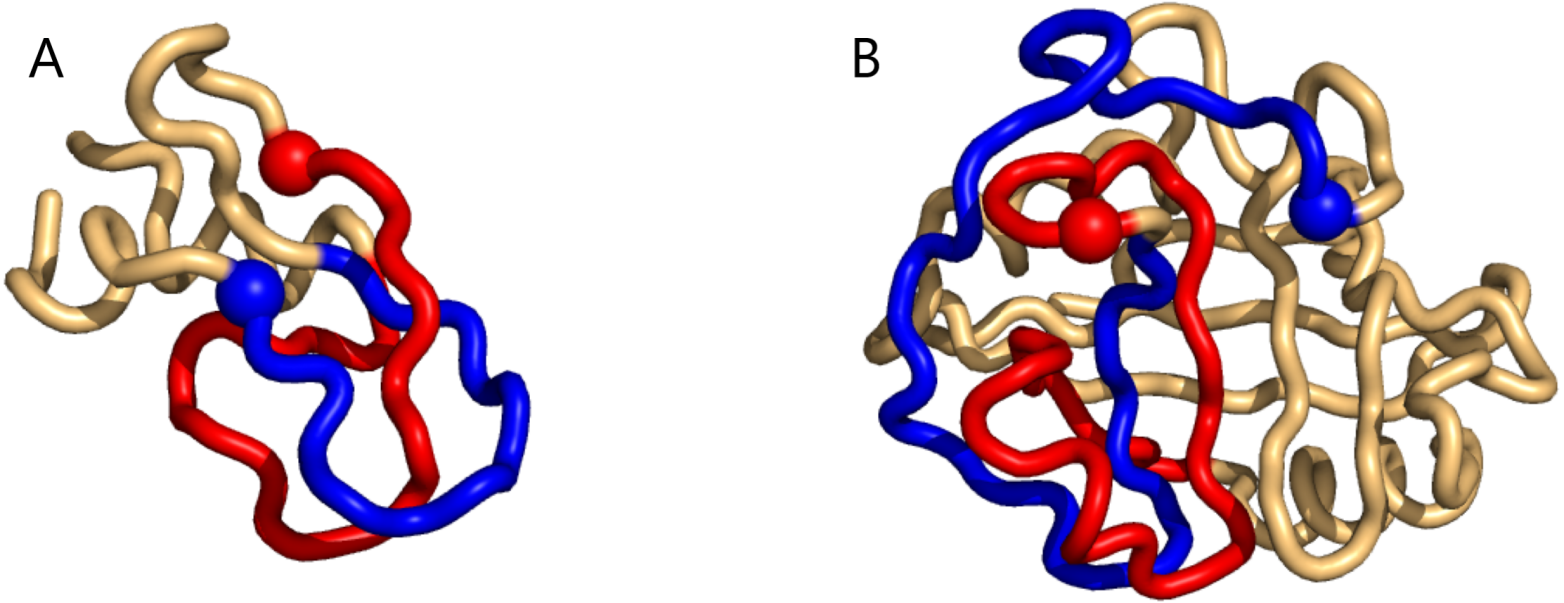
Two examples of particularly high mutual writhe value between the blue and the red sub-chains (the blue is closest to the N-terminus and the spheres indicate lowest residue number in the sub-chain). (A) a 1-link in the rather small 1bpi structure. (B) an example of more elaborate geometry in the 2cpl protein found using the unrestricted search method. The plots were made with Pymol (Pymol, 2016).

In the restricted search we see from Fig. 1A, that the writhe values (almost) fit within the expected interval [−1, 1], and are distributed with rather heavy tails (for top100 and the Pisces sets, see Supplemental Information 1). In the unrestricted search (Fig. 1B), the range of writhe values becomes larger, but values rarely exceed ± 1.5 (less with a preset sub-chain length of 15, see Supplemental Information 1). Notably, in the top100 set, the 1-links found in the restricted search were re-found in the unrestricted search; the two conspicuous cases surfacing from the restricted search through the top100 set were both found among the top 10 absolute writhe value cases in the unrestricted search. However, the restricted search missed a 1-link in the 1dif protein’s B chain, while catching the similar 1-link in the A chain. The reason is that one of the loops in the B chain is not recognized as (almost) closed. While this can depend on the definition of an almost closed loop, the unrestricted search does not have this vulnerability and catches the 1-link in the B chain too. The additional cases of high writhe values (unrestricted search) are in general of more elaborate geometry; an example of particularly high negative writhe in the top100 set is shown in Fig. 2B.

**Figure 3:**
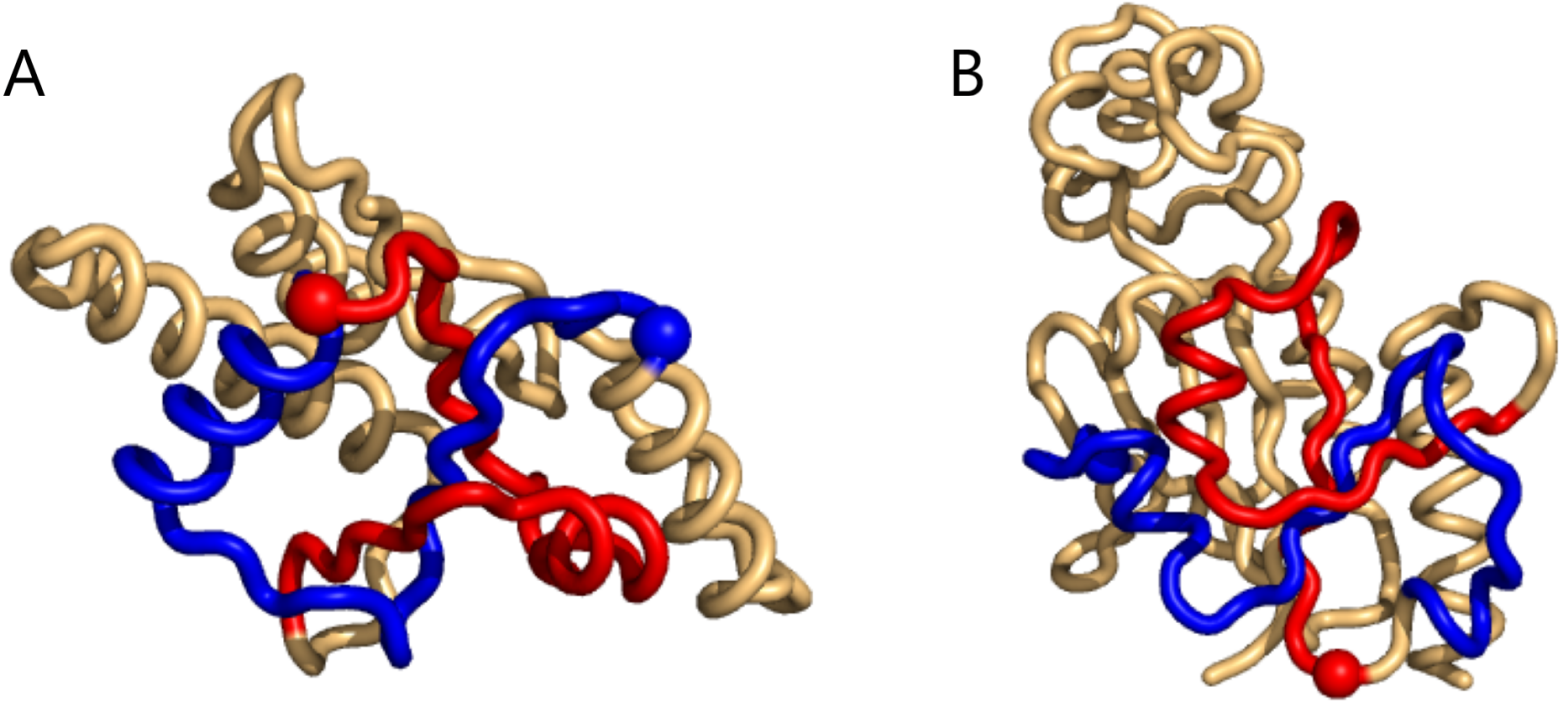
Two cases of particularly high mutual writhe value between the blue and the red sub-chains. These two knots in 1ns5 (A) and 3o7b (B) were found using the unrestricted search method. The plots were made with Pymol (Pymol, 2016).

The same happens for the top8000 set where the 1-link cases are retained, albeit sometimes with lower rank (Supplemental Information 1). In particular on the negative writhe side, the more complex geometries occupy the top ranks and push the 1-links down the list (Supplemental Information 1). As we shall see below, applying GISA’s basic scan tool to the top8000 set, a series of 14 top-negative writhe cases were found, all highly similar to the “pseudo-knot” in Fig. 2B. As for the highest positive writhe, cases appearing to be true knots surfaced (Fig. 3). That is, upon closing a small gap between the sub-chain pairs, if any, the resulting sub-chain becomes a knot (to be rigorous the ends should be “connected at infinity” or similarly).

As for the pokes one may notice that the distributions of writhe values (see Supplemental Information 1) are not heavy tailed as those for the 1-links; indeed “pokedness” is not a binary property as is linking (cf. the electromagnetic analogy above; see also Supplemental Information 1 for a short discussion). Also importantly, less pronounced writhe values reveal uninteresting examples, e.g., two loops well separated; a loop not pierced by the sub-chain (see Supplemental Information 1).

In the PiscesLoRes set the findings are very similar to those in top8000 (Supplemental Information 1). First, the writhe distributions closely resemble each other despite the two sets having a rather small intersection (Figs. S19, S20). It should though be borne in mind that the intersection does not take sequence similarity into account. However, the writhe distributions for the PisceHiRes have the same shapes, a sign that these are really “canonical” of large representative sets of protein chains. The number of structures covered in PiscesLoRes are ∼ 8200 and ∼ 4500 in PiscesHiRes.

In the restriced search in PiscesLoRes we noticed that a single ensemble model, 2q46, dominate at the top of the negative writhe cases (Supplemental Information 1); this can seem peculiar, but the resolution of the model is high.

**Figure 4:**
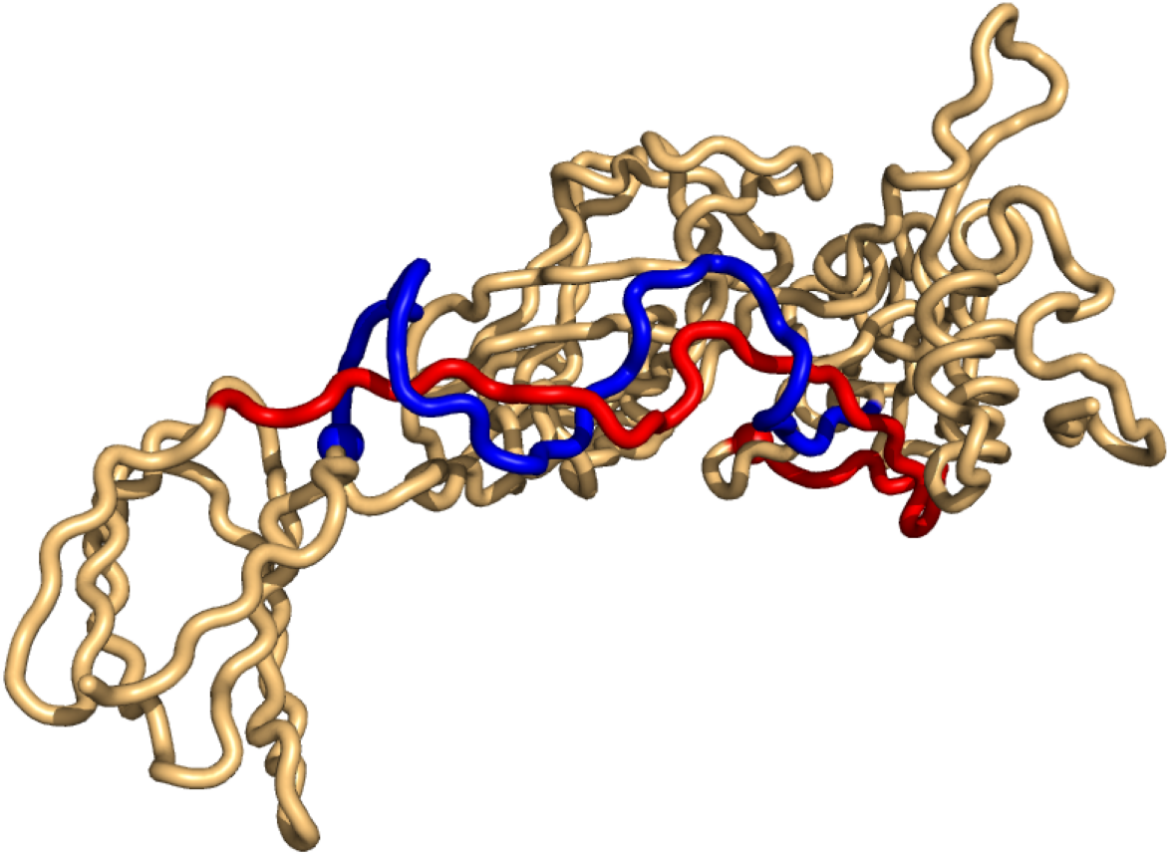
The case of highest negative writhe from the unrestricted search in the PisceLoRes set, 3n40. The plot was made with Pymol (Pymol, 2016).

As for the top-hits in the unrestricted search in PiscesLoRes the cases of positive writhe are all knots (in the same sense as above), while the negative writhe cases are double-pokes in which one sub-chain winds around the other more than 1.5 times (Supplemental Information 1). The most conspicuous case is that of 3n40 where the two sub-chains form a (distorted, long) double-helix of more than two windings (Fig. 4).

Finally, as in the top8000 case, almost all the links of positive writhe are found among the top-hits of the unrestricted search. With the negative cases there is again a tendency of loss-of-rank, i.e., cases of more extreme writhe values are dominating.

### GISA scans

We here consider only the basic scan tool (rar0); validation of the two other tools can be found in Supplemental Information 1. Below we show results from applying rar0 to scans of the top100 set against top8000 as background, and of top8000 against itself. The probability stated in the tables refers to the frequency of structures in the top8000 set having a mutual writhe (or score) of the stated level or exceeding it. For these results a sub-chain (window) length of 30 and a step size of 2 were used. The tables contain excerpts of longer lists found in the Tables S9–S11).

The structures found in the unrestricted search again emerge (as they should, Tables 1 and 2; Tables S3, S11). We also see the clear sign of the skewness of the mutual writhe distributions; in the top100 set the highest negative writhe values appear to be less rare than the similar high positive values. In the ranking from scanning the top8000 set against itself (Tables 3 and 4; Tables S9, S10) this skewness is also clear (cf. also Fig. 1B). Furthermore, the top 10 positive writhe cases (Tables 3; S9) are recorded as true knots in the KnotProt data base (Dabrowski-Tumanski et al., 2018; Jamroz et al., 2015; KnotProt, 2019; Sulkowska et al., 2012) (cf. Figure 3). At the top of the negative writhe list (rank 2–16) are 15 structures of which all except one (2v25) share a very similar structural motif of little secondary structure content (visual inspection; similar to that found in 2cpl, cf. Fig. 2B). Probably as should be expected, these cases are not captured in the KnotProt server (Dabrowski-Tumanski et al., 2018; Jamroz et al., 2015; KnotProt, 2019; Sulkowska et al., 2012) . Since the subchains are very similarly situated in these 14 cases, we ran a multiple sequence alignment using Clustal Omega (Sievers et al., 2011) so as to grasp the sequence similarity and amount of conservation. In the resulting multiple alignment, 20 residues of the about 60 were perfectly conserved, and an additional 10 showed high similarity, an overall similarity of about 50 pct. This though does not appear as high considering the high spatial similarity even of low secondary structural content.

**Table 1:** Top 3 structures in rar0 ranking of the top100 set vs top8000 as background, based on the highest positive mutual writhe pair per structure. Pair refers to the indices of the segments in the chain bordering the two sub-chains.

**Structure/chain**	**Pair**	**Mutual writhe**	**Probability**
dif1/B	(12,42); (64,94)	1.25	2.3 × 10^−3^
dif1/A	(12,42); (64,94)	1.25	2.5 × 10^−3^
1kap/P	(50,80);(102,132)	1.07	8.4 × 10^−3^

**Table 2:** Top 3 structures in rar0 ranking of the top100 set vs top8000 as background, based on the highest negative mutual writhe pair per structure. Pair refers to the indices of the segments in the chain bordering the two sub-chains.

**Structure/chain**	**Pair**	**Mutual writhe**	**Probability**
2cpl	(70,100);(102,132)	−1.75	1.0 × 10^−3^
1nif	(230,260);(260,290)	−1.18	1.8 × 10^−2^
1php	(240,270);(270,300)	−1.04	4.2 × 10^−2^

**Table 3:** Top 5 structures in rar0 ranking of the top8000 set vs top8000 as background, based on the highest positive mutual writhe pair per structure. Pair refers to the indices of the segments in the chain bordering the two sub-chains.

**Structure/chain**	**Pair**	**Mutual writhe**	**Rank**
3onp/A	(60,90);(106,136)	1.50	1
2i6d/A	(164,194);(196,226)	1.47	2
1ual/A	(74,104);(104,134)	1.46	3
1ns5/B	(62,92);(92,122)	1.45	4
3o7b/A	(124,154);(162,192)	1.44	5

**Table 4:** Excerpt of 5 cases in the top15 structures in rar0 ranking of the top8000 set vs top8000 as background, based on the highest negative mutual writhe pair per structure. Pair refers to the indices of the segments in the chain bordering the two sub-chains.

**Structure/chain**	**Pair**	**Mutual writhe**	**Rank**
3hms/A	(0,30);(56,86)	−1.84	1
2r99/A	(70,100);(102,132)	−1.76	2
2wfj/A	(78,108);(110,140)	−1.76	4
1xo7/B	(72,102);(104,134)	−1.72	10
3ich/A	(76,106);(108,138)	−1.68	15

While of relevance in itself, this case also points to using the Gauss Integrals for locally based fold classification or structural alignment. To find such structural similarities by means of superimposing the structures, if at all a viable approach, would be computationally unsurmountable (with about 100 windows in an average structure and 8000 structures it would take ∼10^15^ superpositions). We also noticed other cases where the mere value of the (high) writhe seems roughly to determine the spatial configuration: in the unrestricted search through top8000 at sub-chain length 15 all the top 10 ranking cases (either sign) were of the same “pseudo 1-link” nature (Supplemental Information1).

Finally, regarding the rar0 scan of the PiscesLoRes set against top8000 as background (Tables S12–S13), the results were similar to those for the top8000 set against itself: the top-hits were as in the unrestricted search (Table S8) with some changes in the exact ordering (as for top100, see Supplemental Information 1). Also the same skewness in extreme positive and negative writhe appears as does the skewness in the configurations found: the top positive writhe cases were knots while the extreme negative were rather multiple-wind cases.

### Computational performance

The computational complexity of GISA’s base algorithm for computing the GIs of order less than three is *O*(*L*^2^), while *O*(*L*^3^) in order three (*L* being the length of the chain). When run in order one (and beyond) GISA produces the order one GIs on all sub-chains, where in our implementation we find a run time of ≈2 10^−7^s*L*^2^. The additional time spend on computations done for the proof-of-concept searches amounts to a small overhead of less than 5 % (see section Computational performance in Supplemental Information 1 for these matters).

## Discussion

While there are obvious advantages of using a method not restricted to a sought-after geometry, such as the ability to find new configurations in real proteins and identifying non-protein like ones in models, there is a price to the generality: the unavoidable loss of specificity. In our method this shows up in the loss-of-rank for the 1-links in the unrestricted search through the top8000 set and the PiscesLoRes set, though this essentially only hits the negative writhe cases. However, with a restricted method there is, in addition to its specificity, a likewise unavoidable weakness given by the fact that a definition of the sought-after shapes must be implemented; we saw how one of two highly similar 1-links in the top100 set was missed in the restricted search because a sub-chain was not qualified as a loop.

Regarding the rareness of the shapes (within the considered data sets), the distribution of the writhe for potential links in the top8000 set (Fig. 1A) and the Pisces sets (Supplemental Information 1) are clearly heavy tailed. Among around 1.27 million potential links (top8000), only about 1 out of 100,000 has a writhe below −0.944 or above 0.938, respectively, i.e., there are about 25 such cases in these ∼ 8000 structures. Among the potential pokes in these structures, about 1 out of 10,000 has a writhe below −0.890 or above 0.898, amounting to about 43 of such cases. For the Pisces sets these percentiles have very similar (writhe) values (the PiscesHiRes having slightly fatter tails though). Thus, if we set a threshold for links on absolute writhe of 0.9 (0.95), we find in the top8000 set 31 and 21 (12 and 9) cases having a writhe above the threshold and below minus the threshold, respectively. For the PiscesLoRes these numbers are 26 and 18 (13 and 10) and for PiscesHiRes 25 and 15 (12 and 8). It should be noticed that these numbers relate only to rareness within the top8000 set and the Pisces sets and not to whether these are representative sets of all proteins or not.

For the links these levels compare well to that reported in Khatib, Rohl & Karplus (2009), where 37 links (“co-pokes”) were found in about 10,000 real proteins. Regarding the pokes, the numbers cannot be compared; first poking may be more or less—it is not a binary topological property but rather geometric, and therefore continuously graded; second the method in Khatib, Rohl & Karplus (2009) involves a filter which sifts out most but not all pokes in real proteins. Regarding the results in Dabrowski-Tumanski & Sulkowska (2017), Niemyska et al. (2016) these all pertain to configurations involving loops closed by covalent bonds (e.g., a cysteine bridge), and so are incommensurable to the results reported here. Identifying such configurations rather suggests a separate application of GISA in conjunction with the amino acid sequence.

Our results on rareness are very different from those of Baiesi et al. (2019), where about 1/3 of all proteins were found to be highly entangled. The differences are though not surprising as our results regard local configurations while those of Baiesi et al. (2019) relate to the chains’ global characteristics. We have noticed that the approximation used in Baiesi et al. (2019) for the computation of writhe may have an impact on the decision boundary (threshold) for entanglement (Section S2.2).

The full output of GISA includes besides the writhe of all sub-chains also the remaining 13 generalized Gauss integrals of order at most 2 for each sub-chain. In Røgen & Fain (2003) it was shown that CATH2.4-domains can automatically be assigned to their CATH-fold class based on Gauss Integrals of order at most three of the full domains. The order three GIs are in Røgen (2005) found to be the less descriptive of these structural descriptors. It lies outside the scope of this work to check if the up to order two Gauss Integrals provided by GISA e.g., are sufficient for domain identification and structural classification of all sub-chains, but it is our original motivation for deriving and implementing GISA. KnotProt 2.0 include several methods for topological fingerprinting and can tell either deterministically or statistically if each sub-chain contains a knot or knotoid type (Dabrowski-Tumanski et al., 2018; Goundaroulis et al., 2017). The fingerprints provide data on if and where the searched non-trivial topological features are situated in a given protein structure. Most protein structures are topological trivial (Baiesi et al., 2019) and can therefore not be discriminated based on knot or knotoid content. The GISA output is as a contrast aimed at being a descriptor-vector that can tell members of distinct protein fold classes apart.

As for the computational performance, GISA appears to be efficient and competitive. The free availability of our code makes it feasible to make comparisons of timely performance of other methods to that of GISA.

## Conclusions

We have shown that with the help of GISA it is possible to find cases of rare geometries in proteins, such as those studied in Khatib, Rohl & Karplus (2009) and knots as identified with KnotProt (Dabrowski-Tumanski et al., 2018; Jamroz et al., 2015; Sulkowska et al., 2012). GISA’s command line tool scans formalize this, and more generally scores all the involved structures. The basic rar0 scan corresponds to the approach in the unrestricted search.

The method allows unprejudiced searching, in which other more elaborate shapes are found, while still catching the interesting cases found in the restricted search. Unavoidably, some specificity is lost. As such, the method shows the advantage of quantitative topological fold descriptors. Here the focus has been on applying the lowest order GI (the writhe) and a local search; GISA covers higher order GIs and supports the full range from local to global analysis, which we intend to exploit in upcoming work. In another direction, the two more advanced scan methods can be seen as a foundation for making structural alignments in the space of Gauss Integrals.

##  Supplemental Information

10.7717/peerj.9159/supp-1 Supplemental Information 1 Supplemental Material

## Additional Information and Declarations

### Data Availability

The following information was supplied regarding data availability: The code is available at GitHub (https://github.com/ceegeeCode/GISA).

## References

[ref-1] Baiesi M, Orlandini E, Seno F, Trovato A (2017). Exploring the correlation between the folding rates of proteins and the entanglement of their native states. Journal of Physics A: Mathematical and Theoretical.

[ref-2] Baiesi M, Orlandini E, Seno F, Trovato A (2019). Sequence and structural patterns detected in entangled proteins reveal the importance of co-translational folding. Scientific Reports.

[ref-3] Baiesi M, Orlandini E, Trovato A, Seno F (2016). Linking in domain-swapped protein dimers. Scientific Reports.

[ref-4] Chang P, Rinne A, Dewey G (2006). Structure alignment based on coding of local geometric measures. BMC Bioinformatics.

[ref-5] Dabrowski-Tumanski P, Rubach P, Goundaroulis D, Dorier J, Sukowski P, Millett K, Rawdon E, Stasiak A, Sulkowska J (2018). KnotProt 2.0: a database of proteins with knots and other entangled structures. Nucleic Acids Research.

[ref-6] Dabrowski-Tumanski P, Sulkowska J (2017). Topological knots and links in proteins. Proceedings of the National Academy of Sciences of the United States of America.

[ref-7] Dunbrack Lab (2020). Pisces. http://dunbrack.fccc.edu/PISCES.php.

[ref-8] Goundaroulis D, Dorier J, Benedetti F, Stasiak A (2017). Studies of global and local entanglements of individual protein chains using the concept of knotoids. Scientific Reports.

[ref-9] Jamroz M, Niemyska W, Rawdon E, Stasiak A, Millett K, Sułkowski P, Sulkowska J (2015). KnotProt: a database of proteins with knots and slipknots. Nucleic Acids Research.

[ref-10] Jarmolińska A, Kadlof M, Dabrowski-Tumanski P, Sulkowska J (2018). GapRepairer: a server to model a structural gap and validate it using topological analysis. Bioinformatics.

[ref-11] Khatib F, Rohl C, Karplus K (2009). Pokefind: a novel topological filter for use with protein structure prediction. Bioinformatics.

[ref-12] Kinemage (2016). kinemage.biochem.duke.edu.

[ref-13] King N, Yeates E, Yeates T (2007). Identification of rare slipknots in proteins and their implications for stability and folding. Journal of Molecular Biology.

[ref-14] KnotProt (2019). https://knotprot.cent.uw.edu.pl/.

[ref-15] Marks D, Colwell L, Sheridan R, Hopf T, Pagnani A, Zecchina R, Sander C (2011). Protein 3D structure computed from evolutionary sequence variation. PLOS ONE.

[ref-16] Niemyska W, Dabrowski-Tumanski P, Kadlof M, Haglund E, Sułkowski P, Sulkowska J (2016). Complex lasso: new entangled motifs in proteins. Scientific Reports.

[ref-17] Panagiotou E, Kröger M (2014). Pulling-force-induced elongation and alignment effects on entanglement and knotting characteristics of linear polymers in a melt. Physical Review. E, Statistical, Nonlinear, and Soft Matter Physics.

[ref-18] Panagiotou E, Kröger M, Millett KC (2013). Writhe and mutual entanglement combine to give the entanglement length. Physical Review. E, Statistical, Nonlinear, and Soft Matter Physics.

[ref-19] Panagiotou E, Millett K, Atzberger P (2019). Topological methods for polymeric materials: characterizing the relationship between polymer entanglement and viscoelasticity. Polymers.

[ref-20] Panagiotou E, Plaxco K (2019). A topological study of protein folding kinetics.

[ref-21] Protein Data Bank (2016). RCSB Protein Data Bank. http://www.rcsb.org.

[ref-22] Pymol (2016). The PyMOL molecular graphics system. www.pymol.org.

[ref-23] Røgen P (2005). Evaluating protein structure descriptors and tuning Gauss integral based descriptors. Journal of Physics: Condensed Matter.

[ref-24] Røgen P, Bohr H (2003). A new family of protein shape descriptors. Mathematical Biosciences.

[ref-25] Røgen P, Fain B (2003). Automatic classification of protein structures by gauss integrals. Proceedings of the National Academy of Sciences of the United States of America.

[ref-26] Rose A, Bradley A, Valasatava Y, Duarte J, Prli A, Rose P (2018). NGL viewer: web-based molecular graphics for large complexes. Bioinformatics.

[ref-27] Rose A, Hildebrand P (2015). NGL Viewer: a web application for molecular visualization. Nucleic Acids Research.

[ref-28] Sievers F, Wilm A, Dineen D, Gibson T, Karplus K, Li W, Lopez R, Mcwilliam H, Remmert M, Söding J, Thompson J, Higgins D (2011). Fast, scalable generation of high-quality protein multiple sequence alignments using Clustal Omega. Molecular Systems Biology.

[ref-29] Sulkowska J, Rawdon E, Millett K, Onuchic J, Stasiak A (2012). Conservation of complex knotting and slipknotting patterns in proteins. Proceedings of the National Academy of Sciences of the United States of America.

[ref-30] Zhi D, Shatsky M, Brenner S (2010). Alignment-free local structural search by writhe decomposition. Bioinformatics.

